# Promoting psychosocial wellbeing following stroke using narratives and guided self-determination: a feasibility study

**DOI:** 10.1186/2050-7283-2-4

**Published:** 2014-02-03

**Authors:** Marit Kirkevold, Randi Martinsen, Berit Arnesveen Bronken, Kari Kvigne

**Affiliations:** Research Center for Habilitation and Rehabilitation Models and Services (CHARM) and Department of Nursing Science, Institute of health and Society, University of Oslo, P.O. Box 1153, N-0318 Blindern, Oslo, Norway; Department of Nursing and Mental Health, Hedmark University College, PO Box 400, 2418 Elverum, Norway

**Keywords:** Complex intervention, Nursing intervention development, Psychosocial wellbeing, Stroke, Feasibility study, Multiple case study, Narrative, Quality of life, Patient-centred, Goal-setting

## Abstract

**Background:**

Extensive studies have documented the complex and comprehensive psychosocial consequences of stroke. Psychosocial difficulties significantly affect long-term functioning and quality of life. Many studies have explored psychosocial interventions to prevent or treat psychosocial problems, but most have found modest effects. This study evaluated, from the perspective of adult stroke survivors, (1) the content, structure and process and (2) experienced usefulness of a dialogue-based psychosocial nursing intervention in primary care aimed at promoting psychosocial health and wellbeing.

**Methods:**

This was part of a feasibility study guided by the UK MRC complex interventions framework. It consisted of dialogue-based encounters with trained health professionals during approximately the first year poststroke. It was tested in two formats; individual or group encounters. Inclusion criteria were: Acute stroke, above 18 y.o., sufficient physical and cognitive functioning to participate. Data were collected immediately before, during and 14 days after the completion of the intervention. Pre- and post-data included medical and demographic data, quality of life, emotional wellbeing, life satisfaction, anxiety and depression. Qualitative interviews focusing on participant experiences were conducted two weeks following the intervention. Log notes taken by the health professionals conducting the intervention and work sheets filled in by participants also comprised data. Data analysis was case-oriented. The structured instruments were analysed regarding completeness of data and indication of changes in outcome variables. The qualitative interviews, log notes and work sheets were analysed using thematic content analysis.

**Results:**

Twenty-five stroke survivors (17 men, 8 women), median age 64 (range 33–89), participated. Physical limitations varied from mild to severe. Seven participants had moderate to severe expressive aphasia. The participants found the content and process of the intervention relevant. Both the individual and group formats were found useful. Patients with aphasia reported that there were too few encounters (eight encounters were originally planned). The participants underscored the benefits of being supported through a difficult time, having a chance to tell and (re)create their story and being supported in their attempts to cope with the situation.

**Conclusions:**

This study provides initial support for the usefulness of the psychosocial intervention and highlights areas requiring further consideration and development.

**Trial registration number:**

ClinicalTrials.gov Identifier: NCT01912014

## Background

Psychosocial wellbeing may be threatened following a stroke (Donnellan et al. 2006; Knapp et al. 2000). Depressive symptoms, anxiety, general psychological distress and social isolation are prevalent the first months and years (Knapp et al. 2000; Barker-Collo 2007; Ferro et al. 2009; Hackett et al. [Bibr CR20][Bibr CR21]). Psychosocial difficulties may significantly impact long-term functioning and quality of life (Ferro et al. 2009; Teoh et al. 2009), reduce the effects of rehabilitation services and lead to higher mortality rates (Ferro et al. 2009; Hackett et al. [Bibr CR20][Bibr CR21]).

The causes and risk factors of psychosocial problems are ambiguous. Some researchers theorise that poststroke depression may be a direct effect of ischemic brain lesions damaging the nervous circuits regulating mood (Whyte and Mulsant 2002). However, this theory is controversial (Bhogal et al. 2004; Kouwenhoven et al. 2011). Other researchers assume that poststroke depression is a response to overwhelming stress, affective overload and inability to cope with the extensive losses following a stroke (Whyte and Mulsant 2002; Kouwenhoven et al. 2011). Our work builds on the latter theory and aims to reduce the stress associated with adjusting to the consequences of the stroke by providing psychosocial support and facilitating the stroke survivor’s own coping efforts.

A large number of studies have explored possible interventions for preventing and/or treating psychosocial problems (Knapp et al. 2000; Hackett et al. [Bibr CR20][Bibr CR21]; Forster et al. 2012; Redfern et al. 2006), but the results have been modest. Pharmacological treatment is effective in treating poststroke depression, but not in preventing it (Hackett et al. [Bibr CR20][Bibr CR21]). Psychosocial interventions have had modest effects but indicate that information, emotional support, practical advice and motivational support are important (Forster et al. 2012; Redfern et al. 2006; Ellis et al. 2010). It remains unclear how the different elements of the interventions contribute to positive outcomes and which elements work best at the different stages and among different subgroups (Forster et al. 2012; Redfern et al. 2006; Ellis et al. 2010). Few studies have provided adequate theoretical accounts of the mechanisms assumed to contribute to positive outcomes (Forster et al. 2012; Redfern et al. 2006; Ellis et al. 2010).

In Norway, the context of this study, the municipalities are responsible for providing rehabilitation services beyond the acute phase. However, the municipalities often lack the resources and specialised personnel that are available in hospital-based stroke units. Nurses outnumber specialised rehabilitation therapists in the community care setting, and are therefore more accessible to stroke survivors following discharge from acute treatment and rehabilitation. They are expected to address emotional and psychosocial needs and provide support and guidance to improve coping (Kirkevold 2010). Nevertheless, few nursing interventions have been developed to address the psychosocial wellbeing of stroke survivors (Burton and Gibbon 2005; Forbes 2009; Watkins et al. 2007). Consequently, our goal was to develop a program that can realistically be delivered in the community. In this paper, we report on findings from a feasibility study (Craig et al. 2008) of a psychosocial intervention developed to promote psychosocial adjustment and wellbeing. Specifically, the aims of this study were to evaluate the content, structure and process of the intervention and its usefulness from the perspective of stroke survivors.

### The intervention

**Figure 1:**
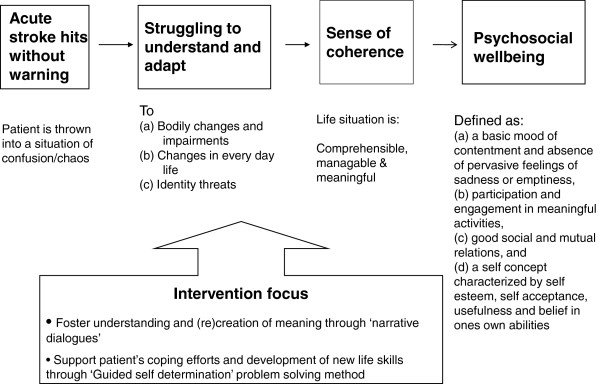
**Theoretical structure of intervention.**

The intervention was developed based on earlier qualitative studies, systematic reviews of psychosocial intervention studies and theories addressing psychosocial wellbeing and coping (for a detailed account, see (Kirkevold et al. 2012)). The theoretical assumptions, guiding the development of the intervention, are summarised in Figure 1.

The overall goal was to promote psychosocial wellbeing, defined as (a) a basic mood of contentment and the absence of pervasive feelings of sadness or emptiness, (b) participation and engagement in meaningful activities, (c) good social and mutual relations, and (d) a self-concept characterised by self-esteem, self-acceptance, usefulness and belief in one's own abilities (Næss 2001). These overarching goals were operationalised in terms of self-assessed satisfaction with each of the domains.

Experiences of chaos and a lack of control are major threats to wellbeing following a stroke (Donnellan et al. 2006; Knapp et al. 2000; Barker-Collo 2007; Ferro et al. 2009; Hackett et al. [Bibr CR20][Bibr CR21]). These experiences are related to difficulties in understanding what is happening in the body, what to expect in the future and how to address the new symptoms, difficulties and a changing life situation. Experiences of chaos and lack of control may threaten the stroke survivors’ sense of coherence (Antonovsky 1987). According to Antonovsky’s theory (Antonovsky 1987), wellbeing is related to a sense of coherence in life (SOC). SOC is promoted by experiencing life events as comprehensible (cognitive), manageable (instrumental/behavioural) and meaningful (motivational) (Antonovsky 1987; Eriksson and Lindström 2005,2006). SOC was assumed to be an essential intermediate goal for promoting psychosocial wellbeing (Figure 1).

To promote SOC, we drew on narrative theory (McAdams 2009; Polkinghorne 1988), which emphasises that human beings create meaning, direction, identity and value in their lives through the stories they tell (Taylor 2007; Kraus 2007). Research suggests that telling one’s story is a fundamental need following a traumatic event and may promote health (Frank 1995,1998). We assumed that being supported to tell one’s story would stimulate reflection and adjustment and would strengthen the identity, self-understanding and self-esteem that are otherwise challenged following a stroke.

People suffering from aphasia are restricted in their natural abilities to tell their stories (Parr 2004; Shadden and Hagstrom 2007). The method “Supported Conversation for Adults with Aphasia” (Kagan et al. 1996) assigns more responsibility to the person who does not have communication difficulties to facilitate social interactions and provides a number of techniques that may enhance communication and understanding in dialogues with aphasia patients.

To promote coping and the development of new life skills, we applied ideas from guided self determination (GSD) (Zoffmann 2004), an approach founded on empowerment philosophy. GSD highlights the importance of being in control of one’s own adjustment process. In this approach, the role of the health care professional is conceptualised as being a “supporter” or “coach” rather than a “care-giver” or “therapist”.

We planned an intervention consisting of dialogue-based encounters between the stroke survivors and specially prepared health care professionals (mostly community care nurses). Dialogue-based was defined as individual or group encounters between equal partners, where the topics and issues of discussion were agreed upon by those involved, based on the needs expressed by the stroke survivor(s). The dialogues were, in principle, open or “unstructured”, inviting participants to voice issues of particular salience at the time of each encounter. However, each encounter had a guiding topical outline that addressed significant issues that are highlighted in the stroke literature as particularly relevant to the stroke trajectory (e.g., bodily changes, personal relations, daily life issues, meaningful activities) (Kirkevold et al. 2012). Each encounter included work sheets developed to support the dialogues. The work sheets consisted of drawings, figures, unfinished sentences and key words pointing to the topic to be addressed (see (Kirkevold et al. 2012; Bronken et al. [Bibr CR5][Bibr CR6]) for examples).

## Methods

### Design

We applied the framework for developing and evaluating complex interventions proposed by the UK Medical Research Council (MRC) (Craig et al. 2008). The MRC framework describes the development and testing of complex health interventions in terms of four major processes; (1) Development of the intervention based on relevant theories and empirical studies, (2) Feasibility testing to evaluate the potential usefulness and methodological issues (3) Evaluation to assess the effectiveness and cost-effectiveness, and (4) Implementation. In our study, we have completed the development and feasibility work. The results from the development work are presented in detail elsewhere (Kirkevold et al. 2012) and are therefore not presented here. However, the reader should keep in mind that the previously published developmental work represents the foundation for the work presented in this paper. In this paper, we present findings from the second phase (the feasibility testing of the intervention), focusing on the stroke survivors’ evaluation and experiences of the intervention. We used a multiple case study approach, wherein each individual participant was studied in detail, drawing on different data sources (Stake 2006). This paper supplements previous feasibility reports from the study, focusing on the aphasia subgroup (Bronken et al. [Bibr CR5][Bibr CR6]) and young persons with stroke (Martinsen et al. 2013).

### Conducting the intervention

The intervention was tested in two formats, as individual dialogues or as a group intervention, with two initial individual encounters followed by six group dialogues with fellow stroke survivors and two group facilitators. Allocation to either individual or group intervention depended on geographical location. At one of the three participating locations, only the group format was offered. At the other two participating locations, the individual format was offered. Twenty stroke survivors received the individual intervention and five the group intervention. The content and work sheets were identical in the two formats. The two individual one-hour encounters that were offered to the group participants were aimed at becoming familiar with the participants' individual situations, establish a working relationship with each participant and addressing early needs before they entered the group sessions. The individual encounters were delivered in a private room in the hospital/rehabilitation unit as long as the participant was hospitalised and in the participant’s home (or nursing home) upon discharge. The group sessions were delivered at a patient education centre associated with a university hospital.

The work sheets were handed out prior to each encounter in order for the participants to be able to review the topics and identify which issues they wanted to discuss at the next encounter. If a participant initially introduced a topic that differed from the topic suggested for the particular meeting, the health care professional was advised to change the planned order of topics, e.g., by using work sheets from other planned meetings. For example, if a participant were very concerned about returning to work or resuming family obligations in one of the first encounters, these topics would be rearranged from later encounters, even if bodily changes had been the planned topic of the day. In this way, the intervention was adapted to meet the individual participant’s needs.

The facilitators delivering the intervention were trained prior to the intervention (16-hour training course) and were supervised throughout the intervention via individual and group supervision sessions. The training consisted of an introduction to the theoretical background and scientific basis for the intervention, the goals and content of the encounters and practical exercises for conducting the dialogues.

The participants suffering from aphasia received individual encounters. A person with in-depth knowledge and specific training in supported communication for persons with aphasia facilitated these individual encounters. The facilitator was supervised by a speech therapist throughout the intervention.

The individual encounters lasted about one hour, while the group meetings lasted 2 hours to allow enough time for all participants to join in the dialogues. For the group format, individual flexibility was more limited. However, the goal was to address issues of common interest among the participants and to allow for discussions of individual needs.

The first meeting occurred as soon as possible after the stroke, usually within 4–8 weeks, and the last occurred approximately 6 months after the stroke (except for the aphasia group, in which the intervention had to be prolonged, see later). The intervention was administered during the period when the adjustment process was assumed to be most challenging (Burton and Gibbon 2005; Watkins et al. 2007; Kirkevold et al. 2012). The meetings were placed at times of increased vulnerability based on known transition points (e.g., at discharge, when physical improvement slows down, assumptions of new challenging roles or activities) (Burton and Gibbon 2005; Watkins et al. 2007; Kirkevold et al. 2012). We developed a guiding timeline suggesting that the first two meetings be carried out prior and immediately after discharge and then every two weeks for about two months and every four weeks the last two months. The timeline was adjusted to meet the needs of the participants in the individual intervention format, but this was not possible in the group intervention due to conflicting needs among the participants. The number of meetings was set at eight in an attempt to balance the ideal with the realistic (i.e. as few encounters as possible but enough to provide adequate support).

### Sampling and recruitment

We chose a purposeful sampling approach. The target group was adult stroke survivors. Inclusion criteria were age 18 and older, having suffered a stroke in the past eight weeks, medically stable, judged by their physician/stroke team to possibly benefit from the intervention, interested in participating, adequate cognitive functioning to participate (judged by stroke team) and speaking Norwegian. Patients suffering from aphasia were included after their language was assessed and specified by a speech therapist. Excluded were persons with dementia and severely ill persons, as judged by their physician/stroke team, for whom the intervention would be of little benefit.

### Setting

Participants were recruited from three different regions in Norway, including two larger cities with large university hospitals and a rural area with two local hospitals and several small counties. The regions were selected to include participants who lived in a variety of urban and rural areas and who received treatment and care from different regional and local jurisdictions. Local recruiters in the hospital or home care service approached potential participants; the recruiters judged whether the patients met the inclusion criteria, provided written and oral information about the study and collected informed consent.

### Data collection

Data were collected immediately before the intervention (T1), during the intervention (T2) and two weeks after the end of the intervention (T3). At T1 demographic data (age, gender, education, job, family relations and living conditions) and medical information (time and type of stroke, functional data, treatment, other medical diagnoses and treatments) were collected. In addition, standardized instruments, measuring health-related quality of life, emotional wellbeing, life satisfaction and anxiety and depressive symptoms were collected (see Table 1). The latter instruments were included to evaluate their appropriateness for a future controlled trial, as the sample size in this study was too limited to conduct sound statistical analyses.

**Table 1:** **Standardized instruments**

Name of the instrument	Type	Concept and dimensions	Scores
Stroke and aphasia quality of life SAQOOL-39 (Hilari et al. 2003 2009)	Health related quality of life (disease specific)	Total score and four sub scores; physical function, communication ability, psychosocial life and energy level.	39 statements where informants rate the extent to which they struggle with the different functions with scores ranging from “can do it” (5) to “cannot do it “(1).
Faces Scale (Andrews and Robinson, 1991)	Global evaluation	Emotional wellbeing	Seven visual faces expressing different degrees of happiness/sadness, with scores ranging from “very happy” (7) to “very sad” (1).
Cantril’s Ladder Scale (Cantril 1965)	Global evaluation	Life satisfaction	Visual ladder with ten steps. Step ten at the top of the ladder depicts the highest level of satisfaction (10), and step one depicts the lowest (1).
Hopkins symptom check list – 8 items (Tambs, 2004)	Symptom specific	Psychological distress/mental health	Eight statements related to common symptoms of anxiety and depression with scores ranging from “not bothered” (4) to “very bothered” (1).

During the intervention (T2), log notes and work sheets were used to describe the intervention process. In the log notes from each encounter, the health care professionals conducting the intervention described their experiences and reflections from each encounter and the reactions and comments from the participating stroke survivors. The log notes were structured to ensure consistency in reporting and focused on the experiences with the content, structure and process of each encounter. The work sheets contained information about the thoughts, feelings, experiences, worries, needs, values and goals that the participants expressed in preparation for and/or during the dialogues.

Two weeks after the intervention (T3), individual in-depth qualitative interviews were conducted, based on a thematic interview guide (see Table 2). In addition, each participant was interviewed using the standardized instruments from T1. The qualitative data represent the data for this paper.

**Table 2:** **Thematic interview guide - qualitative interviews**

Themes	Main questions	Subtopics
Theme 1	Can you tell about how you experience your life at present?	1. Thoughts and feelings regarding present life situation
		2. Psychosocial needs and well-being
		3. Thoughts about the future
Theme 2	Can you tell about your experiences/opinions with regard to participating in the intervention?	1. Number of meetings (too few/too many/appropriate timing of the meetings)?
		2. Length of intervention (appropriate, too short, too long)?
		3. Topics/focus in the meetings (were the topics addressed relevant/were any important topics missing? Was the ordering logical/helpful?)
		4. The worksheets (how did you like using worksheets? What about the content, number, layout, usefulness of the work sheets?)
		5. Inclusion of family/relatives (too little involved, too much involved or appropriate?)
		6. Any advice regarding changes in the content, structure or process of the intervention?
Theme 3	Can you tell whether participating in the intervention has made a difference or not in relation to your well-being?	1. Experiences related to changes in emotional state?
		2. Experiences related to changes in activities?
		3. Experiences related to changes in social relations?
		4. Experiences related to changes self-esteem/identity?
Theme 4	Any other comments/suggestions based on your participation in the intervention?	

The qualitative interview combined open-ended questions with more specific topical questions. The participants were encouraged to describe their experiences in their own words. Some of the persons with aphasia were accompanied by a family member once or several times during the dialogues. In such cases, the family members were also invited to participate in the interview, subject to approval by the participant. Members of the research team, who had not delivered the intervention and whom the participants did not know, interviewed the participants without language problems, allowing them to more openly voice criticism and concerns regarding the intervention. For participants with aphasia, the same person conducted both the intervention and the interviews. Their substantial communication difficulties required continuity in the relationship and familiarity with the intervention process to elicit the participants’ experiences and thoughts. For patients with aphasia, the interviews were video-recorded to preserve as much non-verbal data as possible and supplement their more limited verbal expressions.

### Data analysis

The standardised instruments were analysed qualitatively in terms of degree of completeness of the data and any changes in scores from T1 to T3. A substantial portion of the forms were incomplete and could therefore not be used. For the complete cases, we reviewed the scores in each case in relation to the qualitative analysis to look for consistencies or inconsistencies in terms of expressed experiences. Generally, we found the instruments useful. However, particularly the SAQoL 39 was difficult for some participants to complete, especially at T1, as they expressed that they had not yet experienced many of the activities/situations described.

The qualitative interviews were transcribed verbatim, and the transcripts and log notes analysed with qualitative content analysis (Graneheim and Lundman 2004). We also analysed work sheet notes when these were available (some participants wanted to keep them). The interviews for each case were analysed first. The log notes and work sheets were subsequently analysed using the same approach. The three data sources supplemented each other and gave a richer picture of the participants’ experiences and the nature of the intervention in each case.

The content analysis addressed the following two main questions: 1. How is the intervention judged with regard to the content, structure and process of the intervention? 2. What does the text tell us about the participants’ experiences (positive and negative) of participating in the intervention? The researchers categorized the content of the interviews, log notes and worksheets into subthemes and themes in relation to each of the questions above. At the end, similarities and differences were identified across cases, looking at different subgroups, such as participants receiving the individual versus the group format, participants without language problems versus persons with aphasia, younger participants versus older participants and participants with different degrees of emotional and/or physical challenges. Questions, lack of agreement and unclear issues led to new rounds of analyses until mutual agreement was reached.

### Ethics

The project was reviewed and approved by the Regional Committee for Medical Ethics and the Norwegian Social Science Data Service. Participants provided written, informed consent to a person outside the research group before being included. The consent was adjusted to the needs of persons with aphasia, and they were supported by a speech therapist when necessary. All participants were assured anonymity, confidentiality and the right to withdraw at any time.

### Trustworthiness

Our study confirm to the COREQ criteria (Consolidated criteria for reporting qualitative research) (Tong et al. 2007), which emphasize attention to three domains; the research team, study design and analysis. The last two domains have already been accounted for in the previous sections. With regard to the research team, all researchers conducting this study had a nursing background, were women and were trained as qualitative researchers within nursing science. They had different clinical experiences. Three of the researchers had conducted previous qualitative studies of experiences following a stroke, one specifically focusing on persons with aphasia.

A reference group of multi-professional expert clinicians, researchers in different relevant fields and previous stroke survivors and family members critically reviewed the study protocol and the initial findings, providing significant input.

## Results

### Participants

Of the 29 stroke survivors recruited to the study, 25 completed the study (17 men and 8 women). Four dropped out because of deteriorating health (2), new serious illness (1) or unwillingness to discuss problems (1). The median age of the participants was 64 years (range 33–89). The participants comprised three subsamples; those without language problems receiving individual intervention (13), those without language problems receiving group intervention (5) and persons with aphasia receiving individual intervention (7). The participants’ physical limitations varied from mild (few or no observable mobility limitations) to severe (wheelchair-bound and dependent on assistance for many daily living activities), but most were moderately affected (some muscle weakness and mobility challenges). Several suffered from fatigue, vision or hearing deficiencies, reduced memory and concentration difficulties. Participants with aphasia had moderate to severe aphasia. Twenty-two lived at home, three were discharged to a nursing home following the stroke. By the end of the intervention, one of these had returned home.

In the following, the findings are structured according to the main goals of assessment in this study: (1) assessment of the intervention content, structure and process and (2) assessment of the usefulness as experienced by the stroke survivors. We did not find systematic differences in the experiences recounted between patients with different physical and/or psychological challenges or between participants in the individual and group formats, except from those specified below.

### Assessment of the content, structure and process of the intervention

#### Topics addressed

With one exception (a man with very few limitations following the stroke), the participants judged the topics introduced to be relevant to the experiences, challenges, needs and problems they encountered during the recovery and adaptation processes. Several participants highlighted the importance of addressing the psychosocial aspects of stroke recovery, stating that other rehabilitation professionals taking care of them had not specifically addressed these issues. Some suggested additional topics. The younger participants were concerned about their jobs and economic security, and they talked extensively about their challenges and worries in terms of returning to work. The intervention did not bring up this topic explicitly. Several participants also emphasised the information and support needs of their families and suggested that families be more explicitly included. Many of the participants requested more individualised factual information about stroke treatment and follow-up to help them and their family understand their condition. The following quotations represent the sentiment among the participants:

I (interviewer): What do you think about the content?

P (participant): I think it was fabulous

I: Was anything irrelevant?

P: No! The way it (was)… keep going! (Man 73 y.o., severe aphasia/ individual intervention).

I: Did the topics cover your situation?

P: They did, but there were certain things I missed, like involvement of the family. They have many unanswered questions in a situation like this, but they fall outside, so at least one information meeting for the family was important … And then there are different issues [from patient to patient]. I do not know how clogged my blood vessel was at the time of the stroke, only how it is now. I’d like to know the change, if it is positive or negative, to try to avoid getting it once more. And the medications - what do they actually do and not? (Man, 54 y.o./ group intervention).

#### Work sheets

According to the planned intervention, the participants were expected to review the work sheets prior to each encounter to facilitate individualised dialogues. However, the majority had not done so. Consequently, this part of the intervention did not work as intended. Although they agreed that the topics of the work sheets were relevant, some found the work sheets difficult to understand and use on their own. Some had trouble reading them due to poor eyesight. Others had difficulties concentrating or were afraid that they would provide “incorrect answers”. Some said they just could not make themselves complete the work sheets because of fatigue or simply because they could not write. Others reported that the work sheets were abstract and complex. The following quotations illustrate the participants' experiences with the work sheets:

P: The work sheets were ok to understand, but … difficult to read and write…. The main themes were very good. It was very good that we talked about what had happened and about the future. (Man 43 y.o., aphasia/individual intervention).

P: I don’t think I got that much out of the workbook. I got more out of the conversations with the others. But, then again, I am not a very theoretical person, I am not that good at expressing myself in writing. (Woman, 66 y.o./group intervention).

Those that found the work sheets helpful, explained that the work sheets helped them focus, assisted reflection and led to meaningful dialogues with the health care professionals. Even if they were not able to complete the work sheets themselves, simply examining them helped the participants think through the issues and their relevance. The following quotations illustrate this perspective:

P: [The work sheets] were good. They helped me put things into words. The illustration of the rehabilitation process as a “The Great Trial of Strength” [steneous bicycle race of 500 km] was useful. I have brought this way of thinking about rehabilitation with me. The great trial of strength was quite illustrative. I have travelled from Oslo to Ulven [a very short distance]. The journey has been hard, mainly uphill! (Man, 49 y.o./individual intervention).

P: The content, I think that was very good … to think through the situation that one finds oneself in – I liked that very much.

I: Did you use the work book between the meetings?

P: Yes, I did write to prepare for the meetings … it sort of started my thoughts. (Woman, 33 y.o., aphasia/individual intervention).

#### Number and timing of the dialogues

The participants differed in their opinions about the number of encounters and their timing. None thought that the intervention had too many encounters. Some felt that the timing and number of encounters was adequate and that completing the intervention after eight meetings and six months was reasonable:

P: I think … [the intervention] lasted long enough… (Woman, 66 y.o./group intervention).

P: For me, the number of meetings was just about right. (Man 61 y.o./individual intervention).

Others, particularly the participants with aphasia, felt that the intervention was stopped too early and suggested that the follow-up time ought to be at least one year:

P: [The intervention should last] at least a year, probably two. (Man, 43 y.o., aphasia /individual intervention).

Some felt that although the timing of the meetings was alright, there were too few encounters. They suggested that the meetings should be weekly, at least in the beginning. Particularly among the participants suffering from aphasia, eight encounters were judged to be inadequate. For participants with speech difficulties, the dialogues took much longer and the topics planned for each encounter could not be covered as planned. Consequently, the number of meetings had to be increased, and the intervention prolonged. In the aphasia group, the interventions lasted approximately 10–12 months. The following quotation represents the sentiments among this group of participants:

P: The way I feel, I would have liked more time.

I: Do you mean more encounters?

P: Yes

I: How often would have been ideal for you?

P: Once a week would have been enough, I think.

I: Once a week?

P: Yes, to really master it. (Man, 53 y.o., aphasia/individual intervention).

Each individual encounter was planned to last one hour and the group encounters two hours. The majority of the individual meetings, particularly for participants without speech problems, were completed in about an hour. However, among the participants with aphasia, the time varied widely. Particularly in the early phases after the stroke, the participants tired easily due to their immense struggle in trying to express themselves. The meetings were adjusted individually depending on their stamina and ability to concentrate. The meetings with the persons with aphasia lasted between 40 minutes and 2 hours (one and a half hours on average). The group encounters lasted two hours, as planned.

#### Individual versus group format

The participants were generally positive about the intervention format they participated in, although they differed somewhat in what they emphasised as positive aspects. The participants receiving individual encounters highlighted the importance of their relationship with the health care professional. They stressed the importance of having the same person lead the intervention. Furthermore, they appreciated the supporting dialogues with “a committed professional knowing what they were going through” and the opportunity to discuss issues of personal significance to them:

P: To me, the program [intervention] was luck in an unlucky situation. … That a person has listened to you and kept you under her wings, so to speak, that is good when you are trying to get back into life. (Woman, 82 y.o./individual intervention).

The participants in the group format highlighted the value of sharing experiences and exchange ideas about how to address different issues. However, at the same time, some participants felt that the group format was somewhat restrictive because the dialogues were concentrated on topics that were common between them and less on issues that were individually important:

P: I think it has been interesting, but … the range in age was high, from those that were retired … Whereas I am at a completely different phase of life and had a different stroke (hemorrhage). So even if the treatment is the same, I feel that I have a lot more questions. And I don’t feel that I got answers to them through the project. (Man, 43 y.o./group intervention).

P: I found it very difficult in the beginning because you had to expose yourself. You had to be honest… But when this ”teenager” [young participant] was able to do it … well, it got easier for the rest of us, right? … I think that if I hadn’t had this course [intervention], I would have felt terribly alone. (Woman, 66 y.o./group intervention).

#### Experienced usefulness of the intervention

The experienced usefulness of the intervention highlighted by participants may be classified into three overall themes, as detailed below.

#### Being supported through a difficult time

Many participants considered the intervention to be a highly positive experience and appreciated the access to a series of supportive encounters that they did not have to request. This unconditional offer of support was described as an experience of *not being left alone* in a situation that they experienced as difficult, insecure and scary. They experienced this “going alongside” by a knowledgeable professional as an expression of someone caring for them and providing security:

P: Of course it has helped me along, just knowing you were there and that I could … just move on. … Things are progressing more slowly when you are not here.

I: Do you think it would be helpful with this kind of program for others in a similar situation?

P: Yes, if they get the same [program]. Exactly the same … [getting help to understand] how the stroke affects you … because it is quite strange, being knocked out on one side … and then the way we have talked very well! (Man, 53 y.o., aphasia/individual intervention).

Some of the participants contrasted this positive experience of social support with experiences of feeling deserted by the traditional health services:

P: I didn’t spend many days at the hospital. One day, they came and told me that I was going home. I said no, I can’t go home, we need to talk about rehabilitation somewhere. They gave in that day, but the next day I was “kicked out”, and they left the responsibility for finding a rehabilitation place to the municipality. And then my GP had to help me apply … and I had to call repeatedly to get in. … What I liked with this program was that you followed me up and I didn’t have to do a lot of work to get help”. (Woman, 71 y.o. individual intervention).

Several participants also emphasized the importance of the health care professionals holding up a “vicarious hope” or “vision for the future”, which inspired them to keep on struggling through the difficult times. This facilitated the ‘recovery work,’ when they felt tempted to give up.

#### Provided a chance to tell and (re)create their story

The participants valued the opportunity to tell their stories and talk through their experiences in their own words, supported by the health care provider and the structure that the work sheets provided. Some participants noted that this narrative aspect of the intervention contributed to increasing their understanding of their situation, helped them see possibilities and created opportunities for formulating realistic goals. By talking through their situation, they became more conscious of the different aspects of it. The dialogues helped clarify the issues at stake in their lives and assisted them in reflecting about the possibilities and difficulties. The invitation to tell their stories initiated reflection processes about questions and issues that they had not thought of on their own. Telling their stories also supported their efforts to integrate their experiences and move towards acceptance of the new situation, which happened when the expression of thoughts and dialogue led to reflection and the (re)negotiation of understanding, values and goals. The following quotations encapsulate these experiences:

P: Well, it forced me to think things through … On the one hand, it was good that I had to take a stand. On the other hand – well, it wasn’t exactly exhausting, but it forced me to think things through. And I have had a lot of things to think through – all along… (Man, 49 y.o./individual intervention).

#### Being supported in their attempts to cope with the situation

The participants struggled to cope with their new and unknown situations after the stroke. The issues they struggled with varied widely, from performing daily activities and solving practical problems to understanding and coming to terms with their own emotional reactions and those of their family, friends and colleagues. Facing different social situations within and beyond their family entailed many challenges.

The participants reported that the intervention helped them cope with their struggles. Participants in both the individual and group-based interventions emphasised that the dialogues helped them cope by clarifying what their coping challenges entailed, illuminating their coping options, supporting them as they tried different coping strategies and supporting them as they analysed unexpected situations. Some participants emphasised the importance of being supported in their own initiatives rather than being told how to manage the situation. This led to an experience of being in charge of their lives. The following quotations illustrate these experiences:

P: It has been very good to have someone push me a little – I think it has speeded me up. And then being supported in structuring the days through the work sheet she gave me … (Woman, 71 y.o./individual intervention).

The participants in the group-based intervention also reported that by listening to how other stroke survivors managed their situation, they learned new ways to approach different situations:

P: I always left [the meetings] a little inspired! I think it is important when a serious thing like a stroke happens, that one may exchange experiences with others who have been in the same situation…. That is what has been most important for me – to be together with people in the same situation. The strength of being in a group is that you get to share others’ experiences … I had never realised that you could get psychological problems after stroke unless I had seen one of the other participants … I found that very enriching. (Woman, 66y.o./group intervention).

## Discussion

The major findings in this study was that the participants found the content, structure and process of the psychosocial intervention relevant to their situation and that it contributed with helpful psychosocial support through the initial adjustment process. There were no systematic differences in the experiences and opinions between survivors with different physical and/or emotional challenges or participating in the individual vs. group format of the intervention. In the following we discuss the findings in more detail, relating them to existing knowledge in the field.

### Evaluation of the content, structure and process of the intervention

Our findings confirm that the intervention addresses relevant, concerning issues for stroke survivors. Many of the participants specifically highlighted the importance of addressing psychosocial issues, as they experienced that the existing services did not explicitly address these. Issues of particular salience for many of the participants, particularly the younger ones, were return to work and family obligations and relationships. These are significant issues that may threaten psychosocial wellbeing and should thus receive attention during the adjustment phase following a stroke. Previous studies have found that information, emotional support, practical advice and motivational support are important components for treating stroke victims (Forster et al. 2012; Redfern et al. 2006; Ellis et al. 2010). Compared to these recommendations, our intervention primarily provided emotional support, motivational support and, to a certain degree, practical advice in coping and life skills. The intervention did not include general information about stroke, treatment and follow-up services because we assumed that this information was available through the existing stroke services. However, several of the participants missed individualised information about their stroke to facilitate understanding of their particular situation. This is consistent with other studies on guided self determination (Zoffmann 2004).

The majority of the participants without language problems thought that the number of meetings and the length of the intervention were appropriate. However, the participants with aphasia were unable to complete the intervention within the default time frame. Instead, they required approximately 40% more time to complete the intervention. Determining the number and frequency of encounters was difficult because there is no agreement in the literature. Previous studies of effective psychosocial interventions vary widely on this issue (Burton and Gibbon 2005; Watkins et al. 2007). Based on our findings, it seems important to differentiate between persons with and without language problems when choosing the structure and process of psychosocial interventions, even if the same content is relevant to both groups. Furthermore, our findings suggest that flexibility is needed in terms of the frequency and number of encounters. Because the majority of our participants without language problems found the number of encounters to be sufficient, while some did not, we believe that the intervention should span eight meetings during the first six months as a default. However, for persons still struggling to adjust at the end of six months, additional encounters should be offered. This suggestion is in agreement with Burton and Gibbon’s (Burton and Gibbon 2005) flexible approach. More research is needed to address this issue.

Regarding the use of work sheets to facilitate reflection and dialogue, the findings were inconsistent. Some found the work sheets very helpful, others found them difficult or of little use. This finding is inconsistent with previous studies in diabetes care, which found the use of tailored work sheets useful and efficient in facilitating adjustment and coping (Zoffmann 2004). There are several possible explanations for this finding. First, a stroke entails brain damage, which may affect reading, writing, concentrating and seeing. Although we had considered these consequences when designing the work sheets, emphasizing simplicity and readability, several of the participants found the work sheets difficult. Furthermore, the majority of our participants were elderly, in contrast with the younger participants in the diabetes care study. Older participants may find less benefit and more difficulty in filling in work sheets, and several of our participants expressed worries that they might fill in the sheets incorrectly, although they were repeatedly assured that there were no right or wrong answers. The participants agreed that the encounters were helpful and that the topics introduced by the work sheets were relevant, although they missed some topics (work, family). We conclude that the work sheets have their place in the intervention as an important structuring element but that different participants might utilise them to different degrees.

Testing the intervention in individual and group formats was useful, in that the two forms generated somewhat different experiences and highlighted different challenges. The feasibility study showed that the individual format could be easily adjusted and tailored to individual needs and challenges occurring in the illness trajectory. Because of its flexibility, most participants completed the intervention, and very few missed any of the encounters. However, this is a rather costly intervention with its one-on-one encounters conducted mostly in the participants’ homes. The group format is less costly and provides ample opportunities for sharing experiences with other stroke survivors. However, it is not possible to address individual needs to the same degree as in individual encounters, and the timing could not be as flexible as in the individual intervention. Therefore, participation was not as consistent, and many participants missed one or more of the meetings. These findings are in line with the conclusions drawn in recent reviews (Forster et al. 2012; Redfern et al. 2006; Ellis et al. 2010) and must be considered in the further refinement of the intervention.

### Evaluation of the effective components of the intervention

The major goal of the psychosocial intervention was to promote psychosocial wellbeing by fostering understanding and the (re)creation of meaning, supporting the patient’s own coping efforts and facilitating the development of new life skills. Although the feasibility design did not allow us to evaluate the effect of the intervention on psychosocial wellbeing, the findings suggest that the stroke survivors found the intervention useful. The participants reported that the intervention supported their coping efforts and that this help was needed. They struggled to cope during the first six months and did not experience dedicated assistance with psychosocial issues through the ordinary stroke follow-up services. The participants emphasised the importance of being allowed to tell their story and reflect on their experiences with a knowledgeable dialogue partner. They considered this element of the intervention to be helpful in a situation characterised by insecurity and confusion. In addition to these elements, which we assumed to be effective components (Kirkevold et al. 2012; Antonovsky 1987; Eriksson and Lindström 2005,2006; McAdams 2009; Polkinghorne 1988; Taylor 2007; Kraus 2007; Frank 1995,1998; Parr 2004; Shadden and Hagstrom 2007; Kagan et al. 1996; Zoffmann 2004), the participants also emphasised that being followed up and supported through their own adjustment efforts helped maintain focus and hope as they struggled to reach their goals and resume meaningful activities.

### Stroke survivor support in the community

In Norway, the municipalities are responsible for providing rehabilitation services beyond the acute phase. However, they lack the resources and specialised personnel that are available in hospital-based stroke units. Our intention was to explore whether it would be feasible to conduct an effective stroke support service from the community by giving community care personnel specific, albeit limited, training in the developed methodology. Most of the facilitators in this feasibility study were nurses, who are primary care providers in community care. Based on this study, community-based nurses may integrate this type of follow-up support as part of their responsibility. However, more research is needed to evaluate the effectiveness of this approach.

### Limitations

This feasibility study had several limitations. The sample was limited, particularly in the group intervention part of the study, which may have reduced the variation of experiences and responses. However, because the inclusion criteria were wide, the sample represents a diverse group of stroke survivors in terms of age, gender, level of disability and family and work situations. The participants, including those with aphasia, provided rich descriptions of their experiences through the application of multiple methods and data triangulation. The design did not include a control group; thus preventing us from comparing the experiences of the participants to stroke survivors who did not receive the intervention. Despite these limitations, the case-oriented design and detailed qualitative data from different data sources provided detailed information about the different experiences and viewpoints of the participants.

## Conclusions

This feasibility study provided initial support for the usefulness of the main elements of the psychosocial intervention and provided valuable insights into aspects that require further consideration and development.

## Authors’ information

All authors are nurses with a clinical interest in stroke rehabilitation.
